# The Role of *Streptococcus* spp. in Bovine Mastitis

**DOI:** 10.3390/microorganisms9071497

**Published:** 2021-07-13

**Authors:** Tina Kabelitz, Etienne Aubry, Kira van Vorst, Thomas Amon, Marcus Fulde

**Affiliations:** 1 Department of Engineering for Livestock Management, Leibniz Institute for Agricultural Engineering and Bioeconomy e.V. (ATB), Max-Eyth-Allee 100, 14469 Potsdam, Germany; tamon@atb-potsdam.de; 2 Department of Veterinary Medicine, Institute of Microbiology and Epizootics, Freie Universität Berlin, Robert-von-Ostertag-Str. 7-13, 14163 Berlin, Germany; eaubry@zedat.fu-berlin.de (E.A.); kira.van.vorst@fu-berlin.de (K.v.V.); Marcus.Fulde@fu-berlin.de (M.F.); 3 Department of Veterinary Medicine, Institute for Animal Hygiene and Environmental Health, Freie Universität Berlin, Robert-von-Ostertag-Str. 7-13, 14163 Berlin, Germany

**Keywords:** dairy, cow, mastitis, bovine, streptococci, udder, infection, inflammation, livestock, milk

## Abstract

The *Streptococcus* genus belongs to one of the major pathogen groups inducing bovine mastitis. In the dairy industry, mastitis is the most common and costly disease. It not only negatively impacts economic profit due to milk losses and therapy costs, but it is an important animal health and welfare issue as well. This review describes a classification, reservoirs, and frequencies of the most relevant *Streptococcus* species inducing bovine mastitis (*S. agalactiae, S. dysgalactiae* and *S. uberis*). Host and environmental factors influencing mastitis susceptibility and infection rates will be discussed, because it has been indicated that *Streptococcus* herd prevalence is much higher than mastitis rates. After infection, we report the sequence of cow immune reactions and differences in virulence factors of the main *Streptococcus* species. Different mastitis detection techniques together with possible conventional and alternative therapies are described. The standard approach treating streptococcal mastitis is the application of ß-lactam antibiotics. In streptococci, increased antimicrobial resistance rates were identified against enrofloxacin, tetracycline, and erythromycin. At the end, control and prevention measures will be considered, including vaccination, hygiene plan, and further interventions. It is the aim of this review to estimate the contribution and to provide detailed knowledge about the role of the *Streptococcus* genus in bovine mastitis.

## 1. Introduction

Over 130 pathogens are known to be associated with bovine mastitis [1,2], some of them belonging to the *Streptococcus* genus. Streptococci are gram-positive bacteria of spherical shape (0.5–2 µm) that usually form pairs or chains. They are classified on the basis of colony morphology, hemolysis, and serologic specificity into the Lancefield group taxonomic system. Many of them are facultative anaerobe, non-pathogenic and belong to the commensal microbiota of humans and animals. However, some streptococci can cause severe diseases and health issues, such as bovine mastitis. Here, the most relevant species are *S. agalactiae, S. dysgalactiae* ssp. *dysgalactiae* (hereinafter referred to as *S. dysgalactiae)* and *S. uberis*. Streptococcal pathogens rarely associated with bovine mastitis are *S. canis, S. lutetiensis* and *S. equinus*.

Mastitis is the most common and costly disease in dairy industry and of worldwide relevance [3,4]. The economic losses of mastitis are calculated with 124€ (=147$) per cow per year, resulting in losses of 500 million, 3 and 125 billion € in Germany, the EU and worldwide, respectively [5]. Global bovine mastitis rates are typically between 30–50% of all cows per year [3,6]. Next to the financial losses due to less milk yield and quality, the veterinary treatment, medication, and increased personnel expenses, mastitis is an important issue of animal welfare and the main reason for dairy cow culling. Mastitis infected cows can show a wide range of symptoms: swelling, heat and pain of the udder, milk with abnormal appearance, increased body temperature, lethargy, and anorexia [6,7]. Bovine mastitis can be classified into three classes according to the inflammation degree: clinical, subclinical, and chronic [5]. Clinical mastitis is characterized by visible abnormalities of cow and milk, which is not the case for subclinical mastitis. Here, only the milk yield and somatic cell count in the milk are changed. The incidence of subclinical mastitis is estimated to be 15–40 times higher than for clinical mastitis [8]. Therefore, subclinical mastitis is economically more relevant due to its higher frequency and capacity to reduce milk yields while going unnoticed. The average duration of a streptococcal mastitis is 12 days but can be prolonged for >300 days in chronic cases [9]. If an acute mastitis is not successfully cured, it can become chronic and lead to reduced fertility [4]. *S. uberis* and *S. agalactiae* are well-known pathogens able to induce chronic mastitis [10,11]. Treatment and prophylaxis of mastitis are the most common reasons for antibiotic usage in dairy cows [12,13], bearing the risk of enhanced selection in favor of antimicrobial resistant microorganisms [1,5].

## 2. Classification

Streptococci are reported to be among the main pathogens causing bovine mastitis all over the world [9,14]. Mastitis pathogens can be classified in contagious and environmental [5]. Contagious pathogens are adapted to survive within the host and they spread from cow to cow primarily through the milking process. Contagious bacteria have the potential to spread within a herd easily and widely. In contrast, environmental pathogens are able to survive outside the host and are part of the normal microflora of the cow’s vicinity. Exposure through environmental streptococci occurs during and between milking, during the dry period or prior parturition of heifers [9]. The pathogen exposure is related to their environmental abundance, which is influenced, e.g., by humidity and temperature. Environmental pathogens invade the udder when the teat channel is opened after milking or after damage.

*S. uberis* is primarily environmental, however cases of contagion have been observed [15]. The species is mostly alpha-hemolytic, capable of partial hemolysis, but has also been shown to be non-hemolytic in some cases. Biochemical identification is facilitated by a variable CAMP (Christine–Atkinson–Munch–Peterson test) phenotype as well as aesculin, sodium hippurate and inulin degradation. Global Lancefield classification of *S. uberis* is quite challenging, since some strains have been shown to be Lancefield E, G, P or U positive. Since the first isolation of *S. uberis* from a bovine mastitis case in 1932, the pathogen has been detected in a variety of bovine host infections such as lactating cows, dry cows, heifers, and multiparous cows [15].

For *S. dysgalactiae*, the classification in environmental or contagious is not clear. Due to its ability to survive within the host and in the environment, it is described as an intermediate pathogen [16]. The majority of *S. dysgalactiae* strains are non-hemolytic, although alpha-hemolytic exceptions exist. Phenotypically, it is CAMP negative, does not degrade aesculin and is usually classified Lancefield group C. *S. dysgalactiae* is primarily associated with bovine infections, but other ruminants, such as goats or sheep, may be affected as well.

*S. agalactiae* is a contagious pathogen but may also colonize the gastrointestinal tract of dairy cows. In bovine mammary glands, *S. agalactiae* can survive indefinitely by forming biofilms and is heavily associated with subclinical mastitis [5]. *S. agalactiae* is within the Lancefield group B classification. The bacterium is generally considered beta-hemolytic, but some non-hemolytic strains have been observed and CAMP positive. *S. agalactiae* has nine distinctive serotypes labeled Ia, Ib, II, III, IV, V, VI, VII, and VIII, with a tenth serotype labeled IX discovered in 2007. Pathogenicity of *S. agalactiae* varies with its serotype. Next to dairy, this species is a highly relevant human pathogen at early ages, since it is among the most common causes of bacterial meningitis in neonates. In the United States, between 2005 and 2006, the most common *S. agalactiae* serotype implicated in invasive human diseases was serotype V, accounting for more than 29% of the recorded cases at the time, followed by serotypes Ia, II and III [17]. A myriad of cases have been shown that *S. agalactiae* can be hosted by piscine and aquatic mammals as well. Multilocus sequence typing has allowed the description of the phylogenetic relations between the different hosts of *S. agalactiae* by assigning sequence types (ST) based on their allele numbers. Some strains from piscine hosts were shown to be pathogenic to humans as well. Interestingly, most human STs are quite distinct from bovine STs, apart from the bovine ST-23 and ST-61 strains, which have a genetic relation with human ST-23 and ST-17. These two strains belong to serotypes Ia and III, respectively, and are heavily associated with neonatal infections [18,19,20]. 

*S. canis* belongs to the contagious pathogens and is grouped to Lancefield group G. It shows a beta-hemolytic, CAMP negative, and aesculin hydrolyzing phenotype. *S. equinus* is classified as environmental pathogen and to the Lancefield group D. Its phenotype is a variable hemolysis, CAMP negative and aesculin hydrolysis positive. *S. lutetiensis* is a contagious streptococcal species within the Lancefield D group. It is characterized by alpha-hemolysis and a CAMP negative, aesculin hydrolyzing phenotype.

## 3. Reservoirs, Occurrence and Frequency of Streptococcal Mastitis

As described before, *S. uberis* occurs primarily in the cow’s environment and is the most frequent mastitis causing streptococci with increasing prevalence all over the world [21,22]. The species is detected mainly in the bedding material, at which the contamination of straw was one order of magnitude higher than of sand or sawdust [23]. Lying and bedding areas are the confirmed entry points for *S. uberis* into the udder and are typically characterized by a high bacterial load. Next to *S. uberis*, a very closely related species referred to as *S. parauberis* is existing. *S. uberis* and *S. parauberis* are indistinguishable using phenotypic methods and the only difference is the production of β-d-glucuronidase by *S. uberis* [24,25]. The role of *S. parauberis* for mastitis infections is negligible due to its very low abundance (approximately 0.5% of all mastitis cases).

For *S. dysgalactiae,* the common cattle fly *Hydrotaea irritans* appears to play a significant role in establishment and maintenance of mastitis infections [26].

*S. agalactiae* is primarily occurring within the cow’s udder. However, recent investigations showed that the bovine gastrointestinal tract and the cow environment (floors, bedding and drinking water) are further reservoirs of *S. agalactiae* [5].

*S. canis, S. equinus*, and *S. lutetiensis* are rarely associated with bovine mastitis and were diagnosed in approximately <1% of all streptococcal infections [27,28,29]. For the streptococcal species rarely inducing bovine mastitis, reservoirs and risk factors are largely unknown. *S. canis* mastitis is mainly induced by horizontal pathogen transfer from cats and dogs living in the cow’s environment [30,31,32].

In former times, contagious pathogens were much more problematic than today. In 1927, *S. agalactiae* was considered to be responsible for 90% of all mastitis cases [4,11]. With the introduction of the five-point hygienic plan in the 1960s, frequency of mastitis induced by contagious pathogens was strongly reduced [1,11,23]. This plan included a rapid identification and treatment of infected cows, a whole herd antibiotic dry cow therapy, post milking teat disinfection, slaughtering of chronically affected cows, and routinely disinfection of the milking machine. The implementation of these measures led to a strong overall reduction of mastitis rates (−75%), especially for contagious pathogens as *S. agalactiae* [12]. Currently, the majority of mastitis cases (approximately 90%) have shifted to environmental pathogens [1,33,34]. The proportion of mastitis induced by *S. uberis* achieved more than one-third on a global average [12,23].

**Figure 1:**
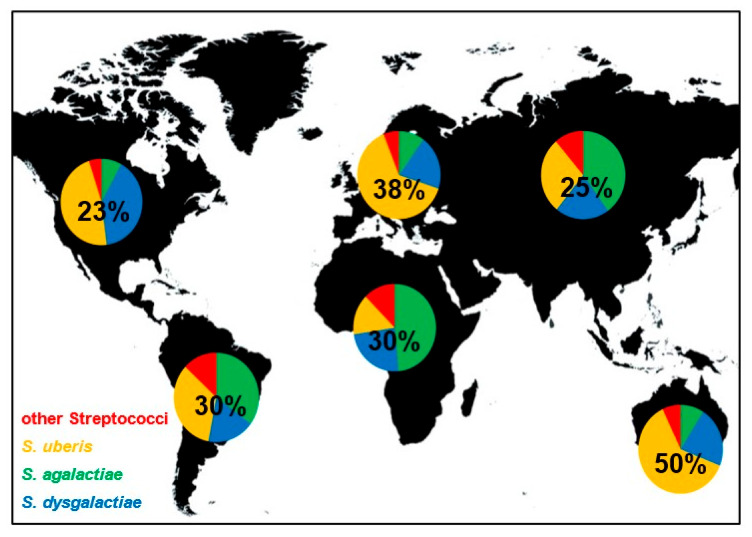
Frequency and distribution of global streptococcal mastitis. Pie charts show the proportion of indicated *Streptococcus* species on streptococci induced mastitis worldwide. Numbers within the pie charts indicate the proportion of streptococcal mastitis of all mastitis cases.

Even if contagious and environmental streptococci induce bovine mastitis all over the world, their distribution is not equal. The worldwide distribution of streptococcal species that induced mastitis is shown in Figure 1. In Europe and Australia/New Zealand, *S. uberis* is causing the most cases of streptococcal mastitis and is estimated to be responsible for approximately 64% and 62% of infections, respectively. In contrast, *S. agalactiae* is responsible for most mastitis infections in Africa and Asia, with 49% and 40%, respectively. In North America, *S. uberis* and *S. dysgalactiae* are the dominant streptococcal mastitis pathogens with similar incidences (47% and 40%). In South America, *S. uberis* and *S. agalactiae* showed highest and similar frequencies (34% and 35%). The proportion of streptococcal mastitis of all mastitis cases is also not equally distributed. Mastitis induced by streptococci seems to be especially frequent in Australia (50%), followed by Europe (38%). On the other continents, *Streptococcus* spp. mastitis are less frequent and responsible for 23–30% of all mastitis infections. Of course, all these values are averaged over large regions and can be significantly different for restricted locations due to environmental factors, local outbreaks, or farm specific practices. However, the data presented in this review give a broad overview about streptococci mastitis distribution worldwide and show differences of broad scale species prevalence.

## 4. Epidemiologic Factors Influencing Susceptibility for Streptococcal Mastitis

Interestingly, there is a large difference between the abundance of streptococci and the number of mastitis cases within and between single herds [9]. It has been shown that several host and environmental factors are influencing the cow susceptibility for streptococcal mastitis. The cow age, lactation stage, parity, and hygienic milking practice were found to have significant influence [35]. Streptococcal mastitis frequency for old cows (>10 years) is approximately 24% higher than for young cows (3–6 years). Medium age cows (7–10 years) showed an intermediate mastitis occurrence. Older cows are more prone to mastitis because of the wider or permanently partially open teat canal as a result of frequent milking [5]. Animals of an earlier lactation stage (month 0–3) have a particularly higher risk of developing streptococcal mastitis than late lactating cows (month 7 and longer) [35]. The lowest risk was observed for dairy in mid lactation stage (month 4–6). The high incidence of mastitis during early lactation phase is supposed due to immunosuppression, associated with the increased oxidative stress and low antioxidant defense [5]. It was observed that the mastitis frequency increases with the number of parities [35]. Cows with a high calving number (>6) developed a streptococcal mastitis 30% more often than cows with few calvings (1–3).

During lactation, the cow has a higher demand of energy and nutrients. It turned out that nutrition influences the immune status and therefore can be correlated with mastitis rates as well. Blood and tissue concentrations of vitamin E and selenium are lowest around calving [9]. Feeding selenium and vitamin E as food supplements reduced the streptococcal mastitis rates [5,23].

The cow susceptibility for mastitis is also influenced by several genetic traits. A positive correlation between mastitis occurrence and milk yield has been identified, meaning that breeding selection for higher milk yields leads to generally more mastitis cases [5,6,8]. Breeding programs selecting for mastitis resistance are mainly focusing on the following properties: a low number of mastitis cases, a low somatic cell count (SCC) in the milk, milk leakage and milking speed. It turned out that SCC is the most useful indirect measure for mastitis resistance and that there is a high genetic correlation between both, which is on average 60–85% [6,8,34]. The teat length, shape, and closure time vary among cows and are further important genetic traits influencing mastitis susceptibility [7,23,36]. Indeed, 70% of the teats were contaminated with environmental streptococci before milking. The teat-end exposure is the major entry point for pathogens [36]. Therefore, mastitis risk is significantly influenced by the teat length, shape, and the time until teat closure completeness after milking and during dry-off period.

The udder microbiota composition and udder mastitis history are two factors that are correlated and seem to play important roles in influencing mastitis susceptibility. The microbiota from udders of healthy cows are clearly distinct from the microbiota of mastitis udders [37]. Healthy udder quarters showed a higher taxonomical diversity compared to ones with mastitis history [38,39]. The results indicate that the microbiota is altered in udders which experienced a mastitis, even if the infection was some time ago. A suboptimal microbiota diversity in udders with mastitis history may be the reason why cows are more susceptible for getting repetitive mastitis [9].

In addition to former listed host factors, mastitis rates were shown to be influenced by environmental factors as well, such as the season of the year and various management practices [9]. Especially the number of contagious streptococci mastitis is in close relation with the milking hygienic conditions. It has been shown that mastitis rates decrease dramatically (−64%) when the udder was washed pre and post milking, compared to no cleaning [35]. Highest mastitis rates are observed in summer compared to spring and winter. Reasons can be the high temperatures and thus the higher proliferation rate of mastitis pathogens in the environment or the transmission of bacteria with flies, which are especially present in summer. *S. dysgalactiae* is known to be transmitted by flies and to be involved in summer mastitis primarily in northern Europe and Japan [26].

## 5. Pathogenesis and Virulence Factors

**Figure 2:**
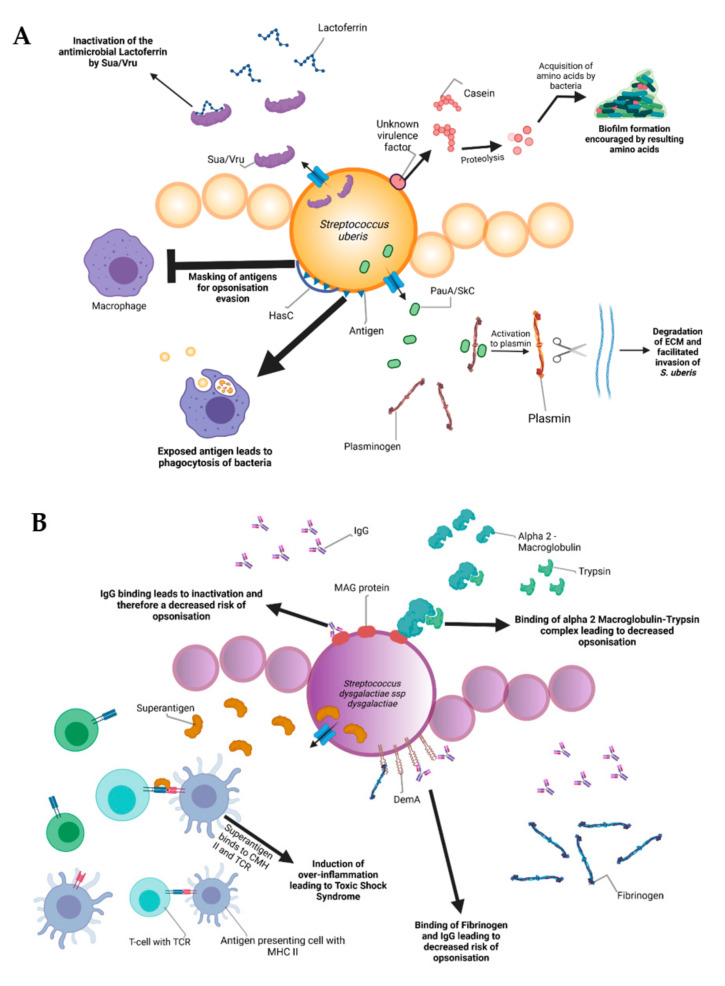

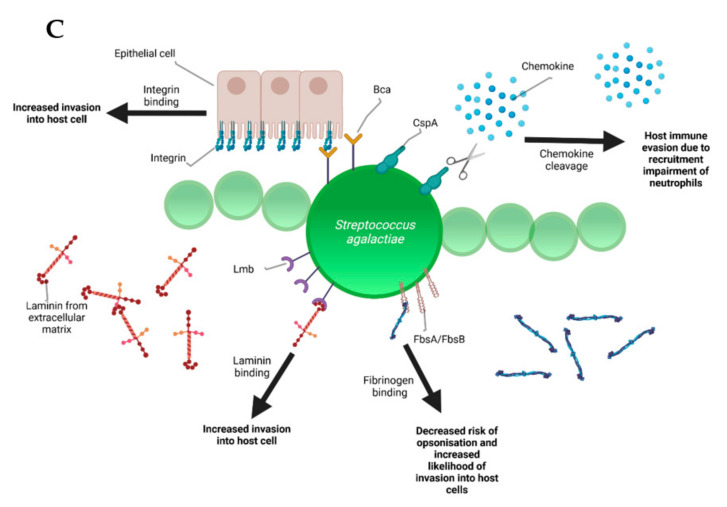
Schematic representation of the bovine-associated streptococcal virulence factors. (**A**) *S. uberis* harbors the capacity for preventing opsonization via the synthesis of the protein HasC, capable of masking the antigens present on the surface of *S. uberis*. Synthesis of Sua and Vru proteins allow binding of the antimicrobial lactoferrin and thus creating a less hostile environment for the pathogen. Lysis of casein through a so far unknown process provides extra nutrients and thereby favors biofilm formation. PauA and SkC proteins confer plasminogen-activating capabilities to *S. uberis*. The activation of plasminogen to plasmin leads to the degradation of the extracellular matrix, which in turn facilitates bovine mammary epithelial cell invasion. (**B**) *S. dysgalactiae* is equally capable of avoiding opsonization via the binding of fibrinogen with the M-like protein DemA, the binding of IgG and the binding of the alpha 2 macroglobulin-trypsin complex via the synthesis of the MAG protein. (**C**) *S. agalactiae* possesses virulence factors implicated in invasion of host cells, namely Bca for integrin binding and Lmb for laminin binding. Immune evasion is facilitated via the synthesis of proteins such as FbsA and FbsB implicated in fibrinogen binding. This leads to a decreased risk of opsonization by phagocytic cells. Synthesis of the serine protease CspA allows *S. agalactiae* to cleave certain chemokines, which are responsible for neutrophil recruitment, further increasing immune evasion potential.

During the infectious process, streptococci face a diversity of changing environments and hence need to adapt to varying conditions for successful infection. As a consequence, they express a number of virulence factors that allow survival and replication in different host tissues during pathogenesis, e.g., by conferring adhesion and invasion to host cells or avoidance of immune recognition and subsequent degradation. The virulence factors of *S. uberis, S. dysgalactiae*, and *S. agalactiae* are summarized in Figure 2.

Amongst all the listed virulence factors, a study of 78 *S. uberis* strains showed that the three most prevalent are the *hasC* gene (hyaluronic acid production) present in 89.7% of the studied strains, followed by the *sua* gene (lactoferrin binding) at 83.3% and *gapC* (plasmin binding as well as immunomodulation) at 79.4%. The *has* operon is a conserved gene region present in all strains of group A streptococci and encapsulated group C streptococci. It enables production of a hyaluronic acid capsule that protects e.g., *S. uberis* from opsonization and phagocytosis and mediates resistance to bacterial clearance within neutrophil extracellular traps [40]. In terms of capsulation, the most well-characterized virulence factors are the capsular polysaccharides coded by the *cps* and *neu* genes, which are at the basis of determining different Group-B-Streptococci (GBS) serotypes discussed later. The capsular types are labeled as Ia, Ib, II-IX and are critical for grouping the different pathogenic types of GBS. These proteins confer immune evasion by masking pro-inflammatory cell wall components and by mimicry of host-cell surface glycoconjugates. SodA and rhamnopolyene also contribute to immune evasion by detoxifying superoxide responsible for the formation of reactive oxygen species implicated in oxidative stress. The C5a peptidase coded by *scpB* is another virulence factor that contributes to immune evasion. C5a is a component of the human complement system, its degradation leads to an inhibited opsonophagocytic killing pathway. In addition to this, the peptidase has been shown to bind to fibronectin, thus contributing to the bacteria’s adhesion and invasion in epithelial cells [41]. 

Internalization of *S. uberis* into mammary epithelial cells is an important early event in the establishment of mastitis in dairy cows. Possession of the *S. uberis* Adhesion Molecule (SUAM), which was suspected to have an affinity to the host protein lactoferrin, seems to facilitate pathogenesis in this context. Lactoferrin is a protein present in human and bovine milk as well as many other mammalian body fluids. The protein has antimicrobial properties as it binds iron, which is essential for bacterial and viral replication [42]. Since iron demand of streptococcal species is rather low, lactoferrin was demonstrated to have a bacteriostatic effect on different streptococci [43]. Clinical trials attempted on seven Holstein Friesian cows showed that 67% of the mammary quarters infected with *S. uberis* wild-type showed clinical manifestations of mastitis (clots in produced milk, firm and moderate swelling of the mammary glands with red coloration). In contrast, experiments conducted with a SUAM-deficient mutant only manifested mastitis in 28.6% of the infected quarters. Colony counts in milk samples as well as somatic cell counts displayed decreased *S. uberis* abundance in the absence of SUAM compared to the wild-type, indicating that the *sua* gene could be an important virulence factor. Moreover, SUAM facilitated efficient epithelial cell adhesion and subsequent internalization [44]. However, other studies have shown that certain factors have overlapping functions with *sua* in terms of pathogenesis of *S. uberis*. Similarly to *sua*, *vru* gene deletion has been shown to decrease the pathogenicity of *S. uberis*, probably due to its capability of binding the antimicrobial lactoferrin [45]. This is an indicator that the *sua* gene should not be considered the only virulence factor of *S. uberis* and that other genes should be taken into account for further research. 

Lactoferrin plays a role in pathogenesis of other streptococci as well. After 8 h of exposure to lactoferrin, a dose-dependent growth inhibition of *S. dysgalactiae* was observed in a previous study due to the antimicrobial properties of the protein. In addition, assays conducted with HC-11 murine epithelial cells revealed lactoferrin-dependent decrease of bacterial adhesion [43]. Lactoferrin may serve as a bridging molecule for *S. uberis*, consequently promoting adherence and internalization into mammary epithelial cells, thereby surviving host defense mechanisms by immune evasion [46]. Although *S. dysgalactiae* also expresses lactoferrin-binding proteins, allowing interaction [47] similar to *S. uberis*, the effect of lactoferrin on *S. dysgalactiae* pathogenesis is quite opposite. The mechanisms of internalization and host cell interaction after lactoferrin binding of *S. dysgalactiae* are poorly defined and require further classification to explain these contrary phenotypes.

Besides lactoferrin-binding proteins, adherence and invasion are also extensively studied for *S. agalactiae*. Exposure of bovine mammary epithelial cells to *S. agalactiae* significantly decreased host cell viability starting 2 h post infection. Electron microscopy showed that adherence of the bacteria was achieved 6 h post-infection with cell membrane breakage visible after 8 h [48]. Furthermore, host-cell adherence and invasion are facilitated by extracellular matrix protein binding. The main virulence factors for this are the fibrinogen binding proteins FbsA and FbsB, which promote entry into the host cell and by the laminin binding protein Lmb. Serine-rich repeat proteins allow for *S. agalactiae* to adhere to human keratin and epithelial cells. The alpha C protein coded by *bca* is also shown to participate in cell adherence to epithelial cells [41]. *S. dysgalactiae* was found to be capable of binding extracellular matrix proteins such as fibronectin, a main component of connective tissues. This could encourage bacterial adherence and internalization, e.g., to the mammary tissue as can be observed in bovine mastitis [49]. Furthermore, binding of *S. dysgalactiae* to vitronectin was implicated in phagocytosis. Exposure of *S. dysgalactiae* to vitronectin before phagocytic assays increased phagocytosis by bovine polymorphonuclear neutrophils [50].

Binding to extracellular matrix proteins is also a key aspect of biofilm formation, which comprises one of the main pathogenic factors of many bacteria and streptococci are no exception. It was previously demonstrated that the addition of milk to liquid THY (Todd–Hewitt broth with 1%, *w*/*v* yeast extract) culture led to an encouragement of biofilm formation by up to 800%. A more detailed look revealed that alpha and beta casein were at least partially responsible for the phenomenon that was decreased by the addition of protease inhibitors. It was speculated that the casein proteins were most likely degraded by proteases, thereby allowing *S. uberis* to utilize the resulting amino acids as nutrient sources [51]. Further studies on *S. uberis* biofilm formation showed that certain genes associated with biofilm formation were more prevalent than others. *luxS* and *comEA* (corresponding to quorum sensing and bacterial competence, respectively) were found at rates of 42.8% and 21.4% of the *S. uberis* isolates which were used for the study. Given the pathogenic capabilities of biofilms, it would be interesting to unravel underlying mechanisms in order to diminish invasive capabilities of *S. uberis* [52]. Furthermore, biofilm formation is suspected to play an important role in *S. dysgalactiae* pathogenesis in cases of bovine mastitis as well. An article by Alves-Barroco and colleagues in 2019 showed that certain strains of *S. dysgalactiae* were capable of forming biofilms on hydrophilic surfaces and that treatment of these biofilms with the protein fisetin induced a decrease in biofilm formation. Fisetin is a flavonol that exhibits various effects, including neurotrophic, antioxidant, anti-inflammatory, and anti-angiogenic effects [53]. Furthermore, a *brpA*-like gene might contribute to the initial steps of biofilm formation. A recent study postulates that this brpA-like protein is inhibited by fisetin, thus rendering the gene a potential target to eliminate *S. dysgalactiae* biofilm formation [54].

Plasminogen activation is yet another virulence mechanism prevalent in the streptococcal genus. Secreted polypeptides, such as streptokinase of *S. dysgalactiae*, can degrade fibrin and connective tissue, thereby allowing deeper tissue infiltration. After exposure to streptokinase, plasminogen is thereby activated to plasmin, in turn allowing the hydrolysis of connective tissue proteins and subsequent tissue penetration [26]. Besides plasminogen degradation, e.g., by streptokinase, *S. dysgalactiae* produces a hyaluronidase, which degrades hyaluronic acid. Hyaluron is a polysaccharide present in connective tissues and its degradation is thought to contribute to the tissue invasive properties of streptococci. The *hly* gene found in *S. agalactiae* is a virulence factor that codes for hyaluronate lyase. The protein is capable of cleaving hyaluronan, allowing for an easier invasion and spread of GBS, similar to the action of streptokinase in *S. dysgalactiae* [55,56]. *pauA* is notable since it was the first described plasminogen activator affecting bovine plasminogen. Furthermore, it encourages the hydrolysis of casein to peptides allowing the bacteria to profit from the resulting amino acids [52]. *skC* is another gene which codes for a plasminogen activator. A study by Loures in Brazil has shown that this gene, along with *pauA,* is highly conserved between different strains of *S. uberis* [57].

*S. dysgalactiae* has a vast array of potential virulence factors, e.g., facilitating binding to diverse host structures that could push towards potential vaccine agents. For instance, *S. dysgalactiae* is capable of binding IgG, therefore interfering with immune responses such as opsonization and toxin neutralization [58]. In addition to IgG binding, a protein named MAG for alpha2-Macroglobulin/Albumin/IgG-binding can additionally connect to alpha2-macroglobulin and is suspected to contribute to the inhibition of opsonization as described previously [59]. It was shown that when alpha2M was associated with trypsin to form alpha2M-T, *S. dysgalactiae* can attach to the complex, thus creating a dose-dependent inhibition of phagocytosis, thereby significantly contributing to pathogenesis [60].

Another notable virulence factor for immune evasion is the serine protease coded by *cspA*. This protease cleaves fibrinogen and chemokines, but also impairs neutrophil recruitment and phagocytic killing. Serotype III of GBS has been shown to be capable of producing a protein called Rib coded by the *rib* gene. Rib is suspected to be one of the reasons for *S. agalactiae* host-immune evasion thanks to a structure that confers domain atrophy (a phenomenon where protein domains lose a significant number of core structural elements) [61]. One final important virulence factor in terms of immune evasion is a superantigen named *S. dysgalactiae*-derived mitogen. Superantigens are a class of antigens capable of inciting an excessive activation of the immune system, thereby inducing potentially life-threatening symptoms such as shock [26].

A versatile variety of other virulence factors facilitate additional essential steps in streptococcal pathogenesis. In terms of bovine mastitis, survival in milk is clearly an important step besides invasion of mammary epithelial cells. The pathogenicity of *S. agalactiae* varies and certain strains are more adapted to bovine hosts in that they are more capable of growing in cow milk than their human or fish-associated counterparts. An article by Maoda Pang and others observed that bovine strains achieved a CFU/mL count of around 5 × 10^9^ as opposed to the 10^8^ CFU/mL of the human and fish strains investigated in this study after 12 h of growth in milk. Furthermore, biofilm formation was more important in bovine-associated strains, showing ODs that doubled or even tripled the ones evaluated in the other strains [62]. SCC indicates the total number of cells per milliliter in milk, which is increased during infection due to the resulting inflammation. According to the 2013 Bulletin of the International Dairy Federation, milk is considered abnormal when SCCs are higher than 200,000 cells/mL [63]. SCCs, determined with an automated, fluorescent, microscopic somatic cell counter, were increased when milk samples contained *S. dysgalactiae*. Indirect fluorescence labeling targeting neutrophils showed that there was a particular increase in neutrophil recruitment [64]. In addition, a notable increase in *Il-1beta* and *TNF-alpha* expression, both being potent inducers of the acute immune response, has been observed via real-time PCR. Further understanding these phenomena could push towards a deeper comprehension of *S. dysgalactiae* pathogenesis [65]. Different virulence traits, however, enable survival of streptococci besides inflammatory responses of the host. For instance, extracellular deoxyribonucleases are shown to aid in bacterial proliferation thanks to the neutrophil-mediated resistance it confers. The enzyme is capable of degrading the antimicrobial system induced by the neutrophils. It was shown in a study by Florindo that all of the *S. agalactiae* strains associated with subclinical or clinical mastitis in 121 clinical samples were capable of synthesizing DNAses [66]. This could imply that DNAse synthesis could be important for the development of bovine mastitis. Further research is required.

M-proteins are the most important virulence factors in streptococcal pathogenesis. It is extensively described in *S. pyogenes* pathogenesis although it is a virulence factor present in a wide variety of streptococcal species. Its best-known property is the capacity to inhibit phagocytosis in non-immune humans. However, it has been associated with a variety of functions and was shown to interact with an extensive amount of host proteins, such as fibrinogen, albumin, plasminogen, and immunoglobulins. The N-terminal part of the protein is hyper-variable, leading to antigenic diversity, consequently allowing efficient serotyping of *S. pyogenes.* The N-terminal region is also known to induce protective antibody production, turning it into a promising vaccine candidate. In contrast, the C-terminal region is highly conserved among M proteins and can bind to a wide array of host molecules [67]. An M-like protein was discovered in *S. dysgalactiae* and *S. canis*, termed DemA and SCM (*S. canis* M-protein), respectively. It displays plasma protein binding properties and sequence similarities with the M-protein of *S. pyogenes*. Given the importance of the protein in Group A *Streptococcus*, it would be interesting to further study the effect of DemA in *S. dysgalactiae* pathogenesis [68].

Group B *Streptococcus* has a huge set of virulence factors that are quite prevalent in most strains isolated from humans. They have been shown to have a wide variety of functions, such as pore-formation via toxins, immune evasion, resistance to antimicrobial peptides, host-cell adherence and invasion. Pore formation via toxins is another main virulence mechanism of *S. agalactiae.* Beta-hemolysin or cytolysins are significant virulence factors, which promote invasion of host cells, but also impair cardiac and liver functions whilst inducing inflammatory responses and apoptosis. The CAMP factor is a pore-forming toxin, which attacks the host-cell membrane and binds to GPI anchored proteins. Resistance to antimicrobial peptides (AMPs) is an interesting virulence mechanism for *S. agalactiae*. Alanylation of lipotechoic acid decreases cell surface charge, thus repelling AMPs. In addition, *S. agalactiae* has been shown to bind penicillin via a protein labeled PBP1a through a so far unknown mechanism. The expression of pili also contributes to AMP resistance, although not much is known about this process either.

Bovine strains possess much of the same virulence factors as the human strains, yet to varying degrees. In an article by Mohammad Emaneini in 2016, it was shown that 89% of the 48 bovine strains expressed the *rib* gene, a value significantly higher than that observed in human isolates, indicating that this gene could be an important virulence factor for bovine-specific cases. However, all of the other virulence factors typically associated with human infections were not detected in bovine isolates. This is in direct contrast with a study by Duarte in 2005, where he showed that the majority of his 38 bovine isolates expressed *scpB* and *bca*. Even the findings concerning the *rib* gene by Emaneini are in direct contrast with a study by Jain in 2012. Jain showed that only 26% of bovine isolates possessed the gene, an incredibly low number, compared to the 89% observed by Emaneini. Overall, determining bovine streptococci mastitis specific virulence factors by comparing those found in human isolates seems to have variable results. The bacteria most likely have bovine-specific virulence factors that are fairly different from those found in human isolates [10,55,69].

Virulence factors for *S. canis* and *S. equinus* are rarely described. For *S. lutetiensis* known virulence factors are: hemolysin (Hly), α C protein (Bca), superantigen proteins, and C5a peptidase (ScpB) [29].

## 6. Diagnosis of Streptococcal Mastitis

The symptoms of a streptococcal mastitis infection are quite diverse for any individual cow and highly pathogen-specific: 43% of cows infected with environmental streptococci showed abnormal milk parameters, 49% showed abnormal milk and a swollen udder, and only 8% exhibited systemic disease signs, such as fever, anorexia, or changes in behavior [9]. Therefore, an economical, rapid, simple, and accurate diagnostic method is essential for mastitis identification and udder health management. There are two phases of mastitis detection: first the recognition that a mastitis is present and second, the identification of the causative pathogen [70]. 

Milk parameters that can be changed due to mastitis are color, consistency, pH, ion concentration, smell, somatic cell number, and the occurrence of flakes or clots [7]. For a long time, the determination of the SCC was the method of choice to identify cows with mastitis [70,71]. Even in the absence of any other disease symptoms, SCC is enhanced in cases of subclinical mastitis. Increase of milk SCC is directly and inversely correlated with milk yield [4]. SCC is an important parameter, because it affects the milk price paid to the farmers. Since 1992, the European standard of milk SCC for human consumption is limited to 400,000 cells/mL [4]. However, mostly a bulk tank milk, SCC < 200,000 cells/mL is recommended for healthy herds and cows [70]. In consequence of a *S. agalactiae* infection, cows SCC is extremely elevated and typically >1,000,000 [72]. SCC can be determined with direct and indirect methods. Directs methods measure the SCC by an automatic cell counting. Laboratory and portable automatic cell counters, for on-farm use, are available [71]. Both systems operate on the principle of fluorescence, where cell nuclei were stained with a DNA-specific fluorescent dye. Another direct detection method is differential cell count. It is performed by laboratory cytometry of milk samples. Recently, an optimized method of differential somatic cell count in individual cow milk samples by means of flow cytometry has been established [73]. Besides total cell count determination and fast mastitis screening, this facilitates a more accurate estimation of the udder health of individual animals. Two indirect and widely used SCC determination methods are the California (CMT) and Wisconsin mastitis test (WMT). The CMT was developed for an easy, cheap and rapid mastitis on-farm detection. Thereby, a detergent (sodium alkyl aryl sulfonate) is added to the milk sample that leads to cell lysis. When the cell number in the milk is above a certain threshold, the sample gets a gel-like viscosity. The WMT is based on the same principle as the CMT but performed in a laboratory with more accurate evaluation possibilities. Therefore, the result is more precise than for the CMT. Wethal et al. (2020) have shown that automated milking systems (AMS) have promising potential for detecting bovine mastitis via milk properties and that the SCC is the most valuable parameter for this purpose [74].

However, until now, measuring the electric conductivity of the milk is the most widespread detection method of mastitis with AMS [75]. During a mastitis infection, the vascular permeability is increased, leading to an elevated level of ions (sodium, potassium, calcium, magnesium, and chloride) and thus higher electric conductivity and pH in the milk [71]. Newest developments in the mastitis recognition through AMS implemented additionally the measurement of milk protease activity as an indicator for mastitis [70].

A mastitis affects many members of the milk proteome and changes their concentrations significantly in comparison to healthy udders [75]. This can be explained by the proteolysis of milk proteins through the pathogens or endogenous proteases. Protein-based milk biomarkers that are well established to detect mastitis are the increased concentrations of the enzymes N-acetyl-β-d-glucosaminidase and lactate dehydrogenase. New mastitis biomarkers under development are the levels of chaperonins for pathogen recognition, prostaglandin D synthase, serotransferrin, bovine serum albumin, various caseins, cytochrome C oxidase, annexin V, haptoglobin and many more [75,76].

After recognizing a mastitis infection, the pathogen identification is substantially important for a rapid treatment and herd mastitis control. Traditional bacterial cultivation in a microbiological laboratory or on-farm is still a widely used method for pathogen identification. Microbial culturing is based on phenotypic identification and biochemical properties of the pathogen, evaluated by growth characteristics, morphology, and ability to metabolize substrates [71]. A smear test of the udders streak canal or more often a milk sample from the beginning of milking is plated on selective growth media. After cultivation conditions are defined, the presence or absence of bacterial colonies is determined. Streptococci grow on classical blood agar media with negative catalase test. Subsequent biochemical and metabolic testing is necessary to determine the streptococcal species (see Table 1 for phenotypic characteristics of bovine streptococci). The major disadvantages of mastitis detection via microbial cultivation are the high rate of false negative results (27–50%) and the time consuming (24–48 h) and labor-intensive analysis [70]. In approximately 30% of mastitis cases, traditional phenotypic methods fail to identify the disease, because only viable pathogens can be detected.

Next to bacterial cultivation, mastitis pathogen detection can be performed by molecular diagnostic methods, such as mass spectrometry, immunoassays, and DNA-based genotyping [71]. Protein-based mass spectrometry is the optical identification of pathogen-specific peptides by their weight and correlated time of flight. A mass spectrometry study of [27] identified three biomarkers specific for bovine streptococci with peaks at m/z values of 2550, 2634, and 2650. The identification of the causative streptococcal species was possible with specific other peaks as well (*S. agalactiae*: 2112, *S. dysgalactiae*: 5955, and *S. uberis*: 4452). Immunoassays (ELISA) use specific antibodies against mastitis pathogen surface structures, but have been mainly developed only for *Staphylococcus aureus* and *Escherichia coli* [75]. Genotyping methods use DNA for the identification of mastitis pathogens as streptococci. A popular and well-established method for the direct detection of infectious agents is PCR or PCR-based methods (e.g., microarrays, next generation sequencing, fluorescence in-situ hybridization, restriction fragment length polymorphism, amplified fragment length polymorphism, etc.). Up to 30% of clinical mastitis samples do not grow on conventional cultivation media and therefore will not be detected [37,71]. Because PCR-based analyses are cultivation independent, rapid and highly specific, they are especially suitable for such samples. 

In the field of mastitis diagnosis, the importance of behavioral changes should not be ignored. If etiological changes in correlation with a mastitis appear, they are already evident a few days before clinical symptoms occur [70]. Therefore, behavioral abnormalities can be used as sickness indicators and can be recorded with a sensor-based automated health monitoring system. Suitable parameters that can be measured with sensors are the rumination activity, physical activity, body and udder temperature, rumen pH, respiration rate, water and feed intake and heart frequency. Udder temperature measured with thermal and infrared cameras was shown to be 1–1.5 °C higher for cows with mastitis [75].

## 7. Conventional and Alternative Therapy Strategies

Mastitis is a complex and multifactorial disease. Therefore, its therapy is not trivial. Antibiotic therapy is the main strategy for mastitis treatment [4,13]. The milk ducts and alveoli of the mammary gland are primary targets of the antimicrobial therapy against streptococcal mastitis. β-lactam antibiotics, such as penicillin, oxacillin and ampicillin, are the most effective and most applied antibiotic class to treat mastitis caused by streptococci [24,33,77]. Antimicrobial treatment is usually administered by intramammary syringe or parenterally by intramuscular injection [23,36]. Both application forms are comparably effective. The intramammary infusion should be preferred instead of muscular application, because it needs significantly less drugs and it avoids the systemic distribution of antibiotics within the cow. Pathogens as *S. uberis* and *S. agalactiae*, which are able to reside intracellularly within the mammary gland and to form abscesses, are more difficult to treat due to the restriction of their contact with antibiotics [33,78]. Such infections are predestinated to become chronical. The earlier the antibiotic therapy is carried out after appearance of mastitis symptoms, the higher are the cure rates [23]. Intramammary antibiotic therapy has obtained cure rates of approximately 90% for subclinical mastitis caused by *S. agalactiae, S. uberis,* and *S. dysgalactiae* and 77% for other streptococci [33]. The high use of antibiotics in dairy husbandry represents a serious problem related with the emergence of antibiotic resistant bacteria and antibiotic residues entering the food chain [13]. Almost 90% of antibiotic residues detected in milk originate from mastitis therapy [33]. Thus, there is urgent and extensive research to find non-antibiotic therapy alternatives.

One of these alternatives is the bacteriophages therapy. For mastitis treatment, it will be necessary that specific bacteriophages are transferred into the cow’s mammary gland and remain active and stable when they come into contact with raw milk. This seems to be the major challenge, because previous studies observed an inhibition of bacteriophage K against *Staphylococcus aureus* by milk [79,80]. To our knowledge, all research of mastitis phage therapy so far concentrated on *S. aureus* control. There is essential need for the investigation of phage therapy against streptococcal mastitis infections. 

Over the last years, nanoparticles drew attention as antimicrobial agents of low toxicity.

Metal nanoparticles of silver, gold, or copper are bactericidal by damaging cell membranes, protein denaturation, DNA degradation and the formation of reactive oxygen species [34,81]. Gram-positive bacteria as streptococci are especially sensitive to copper nanoparticles. Amoxicillin nanoparticles were biologically active against *S. agalactiae* in vitro [82]. In addition to the low price, safety NO (nitric oxide)-nanoparticles can be an alternative to antibiotic treatment [13]. 

Another alternative for antimicrobial therapy against mastitis is anti-inflammatory therapy. Thereby drugs (e.g., dexamethasone) inhibit the synthesis of inflammatory signal molecules, which include glucocorticoide, leukotrienes, prostaglandins, and thromboxanes [33]. So far, the effectivity of anti-inflammatory therapy against mastitis is controversial, probably due to the complexity of the inflammation process. Anti-inflammatory drugs may provide symptomatic relief and promote well-being on the part of the affected cow, but they do not eliminate the reason of infection.

Bacteria-derived antimicrobials (bacteriocins) present a potential option to replace antibiotic therapy. Metabolites of lactic acid bacteria demonstrated an antimicrobial activity against *S. uberis* and *S. agalactiae* in vitro [83]. Nisin, an antimicrobial peptide produced by *Lactococcus lactis*, has been shown to be comparably effective against mastitis pathogens as antibiotics [81]. A Canadian and Polish study confirmed a high nisin efficacy against all streptococcal mastitis pathogens, even though the nisin concentration was low (19.5 IU*mL^−1^) [14,84]. In some countries, nissin in form of wipes is used for pre- and post-milking teat disinfection (product: Wipe Out^®^).

Animal-derived antimicrobials are mostly immunomodulatory proteins produced by mammals. Lactoferrin and β-Lactoglobulin are two antimicrobial proteins present in milk, which showed significant inhibitory effect against *S. agalactiae* and *S. uberis* in vitro [85,86]. Lysozyme is an animal protein contained in milk, saliva, serum, and eggs, which has a hydrolyzing effect on the peptidoglycan part of bacterial cell wall. It has been used successfully to increase the antibiotic efficacy against mastitis induced by *S. uberis* and *S. dysgalactiae* [87].

Plant-derived antimicrobials showed a high promising potential to counteract mastitis pathogens and a high number of diverse herbal extracts with antimicrobial activity are described [5]. Their main advantage compared to antibiotics is that they do not induce resistances even after prolonged exposure [81]. Cinnamaldehyde, eugenol, carvacrol, and thymol exhibited antimicrobial activity against *S. agalactiae, S. dysgalactiae* and *S. uberis* [88]. Cinnamaldehyde was the most effective substance and reduced the streptococcal pathogens in milk from10^4^ –10^5^ colony forming units per mL to undetectable levels within 12 and 24 h. Further studies successfully tested 7-epiclusianone from *Rheedia brasiliensis,* Copalic acid, a diterpene from *Copaifera langsdorffii* (diesel tree), extracts from *Tabernaemontana divaricata* (pinwheel flower), and palm oil for their antimicrobial activity against mastitis streptococci [89,90,91,92]. Montironi et al. (2016) have shown that essential oil from *Minthostachys verticillata* and limonene have an inhibitory effect on *S. uberis* activity and biofilm formation. However, there are numerous more plant-derived antimicrobials that can serve as alternative or complementary therapy for bovine streptococcal mastitis [93].

Concluding, numerous therapeutic alternatives with promising in vitro results to treat bovine mastitis exist. Plant-derived antimicrobials showed the most encouraging results so far. However, at the moment, no antibiotic alternative for in vivo usage is available [94]. Due to the knowledge that raw milk and its components can negatively impact the antimicrobial activity of diverse agents, in future, it will be necessary to evaluate the antimicrobial performance of alternative therapeutics in milk and in vivo. Therefore, the replacement of classic antibiotic therapy seems to be realistic, but likely not in the near future.

## 8. Antimicrobial Resistances (AMRs) in Bovine *Streptococcus* spp.

As mentioned above, the most common treatment of streptococcal mastitis is a β-lactam antibiotic therapy. The extensive use of antibiotics in dairy husbandry generates an increased risk of emerging AMR microorganisms that may then enter the food chain and affect human health [1,13,94]. Data about AMR in bovine streptococci vary massively depending on the streptococcal species, geographical location, study design (sampling size and scheme, method for resistance determination) and literature source [95,96,97,98,99,100,101]. Here, we aimed to present representative studies about AMRs in bovine streptococci with a meaningful study design. 

A very recent and extensive study from France identified that antibiotic resistance levels of *S. uberis* were low for oxacillin (on average 2.2%) and gentamycin (2.4%), and attained 9.3% for trimethoprim-sulfamethoxazole (SXT), whereas resistance levels to tetracycline (18.1%), lincomycin (19.1%) and erythromycin (20.0%) were ever higher [102]. The highest resistance level of *S. uberis* was found to enrofloxacin with 32.9%. Resistance rates for the antibiotics tested were more or less stable over 10 years, except for tetracycline resistance that showed a linear increase between 2006 and 2016 from 15.7% to 20.4%. Further, 15% of *S. uberis* isolates were characterized to be multidrug resistant to three or more antibiotic classes [102,103].

*S. dysgalactiae* seems to behave a bit differently concerning antibiotic resistances. For this species, the highest proportion of resistance was identified against tetracycline (38.5%), followed by much lower resistances against erythromycin, lincomycin, kanamycin, enrofloxacin and streptomycin (3–5%) [12]. Oxacillin and gentamycin resistances were almost not found (0.7% and 0%, respectively). Multidrug resistance for *S. dysgalactiae* was determined to be 6% [103].

*S. agalactiae* showed the highest resistance rate against sulfatrimethoprim (50.5%) and tetracycline (46.2%), followed by erythromycin resistance of 15.4% [33,104]. Resistance rates against ampicillin, penicillin and pirlimycin were 2.6%, 3.9% and 7.1%, respectively. No resistance against lincomycin was reported [105]. The higher prevalence of tetracycline resistance in *S. dysgalactiae* and *S. agalactiae* compared to *S. uberis* (Table 1) is a global trend and has been detected by several resistance monitoring programs [105]. In general, antibiotic resistances of *S. agalactiae* seem to be a bit higher than of *S. uberis* and *S. dysgalactiae.*

*S. canis* isolated from mastitis cows showed no resistance to cephalothin and oxacillin, but 33% showed an intermediated penicillin resistance [28]. For *S. lutetiensis*, the highest antimicrobial resistance (63%) was detected for enrofloxacin [29], followed by ceftiofur (49%), tetracycline (43%), erythromycin (24%) and penicillin (9%). Further, 24% of all S. *lutetiensis* isolates were multidrug resistant and all were susceptible to vancomycin.

Overall, antimicrobial resistances in mastitis-causing streptococci are abundant and slightly increasing [94]. The degree of resistance is strongly dependent on the streptococcal species, antibiotic type, location, and herd. In general, the dynamic of resistance acquisition in streptococci is slower than what is experienced in *Enterobacteriaceae*, probably due to much lower rates of horizontal transfer of resistance genes [105]. Summarizing all streptococcal mastitis species, tetracycline resistance is the most common, followed by resistance to erythromycin, pirlimycin and gentamicin [103]. Interestingly, even though streptococcal mastitis is typically treated with β-lactam antibiotics, streptococci are still very susceptible to them [106]. Therefore, penicillin and its relatives remain the antibiotics of choice for streptococci-induced mastitis.

## 9. Control and Prevention

Preventing a disease is always better than treating it. The risk factors for the different streptococcal species to cause mastitis are pathogen-specific (Table 1) [107]. For decades an effective vaccine for the prevention of bovine mastitis has been searched [94]. So far, vaccine development focused on *Staphylococcus aureus*, *S. uberis,* and *Escherichia coli,* but with limited efficacy [13,36]. Reasons are an ineffective adjuvant formulation, an improper immunization schedule and a non-satisfying range of protection against different strains. For a long time, the development of a *S. uberis* vaccine has been ongoing, but to date, there is no commercially available vaccination against streptococci [23,36]. A subunit vaccine against the plasminogen activator PauA of *S. uberis* has been developed and conferred a partial protection of 38% and 63% from clinical mastitis, depending on the added adjuvant [108]. Repeated subcutaneous injection with killed *S. uberis* cells reduced the pathogen proliferation, but mastitis still occurred [109]. A recent trial developed a subunit vaccine called UBAC (Hipra, Amir, Spain) with lipoteichoic acid from the ‘biofilm adhesion component’ of *S. uberis* [36,110]. It reduced the clinical mastitis signs and the bacterial counts, but was not able to prevent the disease. Cows immunized with the cell surface-associated GapC protein of *S. uberis* showed a significant reduction in inflammation severity, but showed no reduction when immunized with *S. agalactiae* GapC, suggesting there is no cross-species protection [111].

New mastitis infections occur particularly often during the cow dry-off period, more often than to any other time point of lactation [1]. Environmental streptococci mastitis rates were 5.5-fold higher during the dry period compared to the lactation period [9]. The high mastitis rates during the dry period may be reasoned by the lack of teat flushing through milking, changes in the mammary secretion and microbiome and the possible absence of the teat keratin plug. 90% of all dairy cows are preventively treated with antibiotics during the dry-off period (called blanked dry cow therapy) and this is an essential part of the mastitis prevention program. Dry-cow antibiotic therapy is supposed to be the most economical and efficient method of treating and preventing streptococcal mastitis [23,72]. However, concerns about the high usage of antibiotics are increasing [5]. Recent studies have shown that teat sealant instead of antibiotic therapy can be comparably effective to dry-cow therapy [78]. The teat sealant (bismuth subnitrate paste) creates a physical barrier that helps to prevent entrance of pathogens into the mammary gland. The mean abundance of streptococci in the udder of dry-off cows was even lower with the teat sealant than with antibiotics. Use of teat sealant during the dry period resulted in a similar incidence of mastitis, somatic cell count, bacterial load and milk microbiome to antibiotic therapy.

The basis of all preventive methods for streptococcal mastitis is limiting of teat end exposure. Housing facilities and management practices contribute to that [9]. Therefore, lying and bedding areas should be clean and especially dry, avoiding deep straw packs. Ventilation is critical to maintain dry conditions and frequently poor in older facilities. As bedding material, sand or sawdust should be preferred instead of straw [23]. To prevent mastitis through environmental pathogens, it is advised that the cow stands for at least one hour after milking until teat canal is properly closed. In general, pastured cows have a reduced risk of getting infected by streptococcal mastitis compared to housed cows [9]. However, also in pastures, conditions advantageous for environmental streptococci proliferation exist, e.g., areas under shade trees, over grazed pastures, or pastures with high soil moisture.

Infected cows should be rapidly detected and separated to avoid bacterial spreading within the herd [72]. The bacterial contamination of the individual cow should be lowered by good milking practice, such as teat disinfection and drying, as well as regular cleaning and checking of the milking machine. The application of these measures reduced the incidence of streptococcal mastitis by 50% [9].

## 10. Conclusions

Historical evidence suggests that cows have been domesticated and milked since at least 3100 BC and it is likely that bovine mastitis has existed since that time. Even after thousands of years and intensive research regarding mastitis, it is still an important economic and animal welfare issue and the most frequent and costly disease in dairy farming. Streptococci are at the forefront of mastitis isolates and account for 25–50% of global mastitis cases. In countries and areas with good hygienic milking practices, contagious streptococcal mastitis has been strongly reduced since the 1960s. However, in Africa and Asia, it is still a serious problem. In the rest of the world, environmental streptococci are dominating, causing 40–70% of streptococcal mastitis. Antibiotic resistances are increasing at an alarmingly high rate, emphasizing the need for alternative therapeutic measures. In order to develop new preventive strategies or therapeutics apart from antibiotic interventions, a comprehensive understanding of the pathogenesis of streptococci and the virulence factors involved in different mechanisms upon host cell infection is of crucial importance. Here, we summarized traits contributing to the epidemiological spread of common streptococcal mastitis isolates and described differences and similarities in host cell interaction, infection, and pathogenesis.

## Figures and Tables

**Table 1:** Overview of *Streptococcus* species causing bovine mastitis.

*Streptococcus* Species	Lancefield Classification	Transmission	Phenotype (+ Positive − Negative)	Virulence Factors	Antibiotic Resistance (Top 3)	Risk Factors	Host Specificity
*Streptococcus* *agalactiae*	B	contagious	beta-hemolytic + CAMP − aesculin	CspA Bca FbsA/FbsB Lmb	50.5% sulfatrimethoprim 46.2% tetracycline 15.4% erythromycin	milking	zoonotic
*Streptococcuscanis*	G	contagious	beta-hemolytic − CAMP + aesculin	SCM SLS	33% penicillin 0% cephalothin 0% oxacillin	pathogen transfer from cats and dogs	zoonotic
*Streptococcus* *dysgalactiae ssp.* *dysgalactiae*	C	intermediate	non-hemolytic − CAMP − aesculin	MAG DemA Superantigen	38.5% tetracycline 4.8% erythromycin 4.4% streptomycin	summer (transfer by flies)	cattle- specific
*Streptococcus* *equinus* (previpously *S.* *bovis* type II/2)	D	environmental	variable hemolytic − CAMP + aesculin				zoonotic
*Streptococcus* *lutetiensis* (previous *S.* *bovis* type II/1)	D	contagious	alpha-hemolysis − CAMP + aesculin	Hly scpB Bca Superantigen	63% enrofloxacin 49% ceftiofur 43% tetracycline		zoonotic
*Streptococcus* *uberis* *(Streptococcus* *parauberis)*	diverse (mostly E)	environmental	alpha-hemolytic variable CAMP + aesculin	Sua/Vru HasC PauA/SkC	32.9% enrofloxacin 20.0% erythromycin 19.1% lincomycin	straw as bedding material	cattle- specific

## Data Availability

Not applicable.

## References

[1] Bradley A.J. (2002). Bovine Mastitis: An Evolving Disease. Vet. J..

[2] Leigh J. (1999). Streptococcus uberis: A permanent barrier to the control of bovine mastitis?. Vet. J..

[3] Hogeveen H., Huijps K., Lam T.J. (2011). Economic aspects of mastitis: New developments. N. Z. Vet. J..

[4] Ruegg P.L. (2017). A 100-Year Review: Mastitis detection, management, and prevention. J. Dairy Sci..

[5] Cheng W.N., Han S.G. (2020). Bovine mastitis: Risk factors, therapeutic strategies, and alternative treatments—A review. Asian Australas J. Anim. Sci..

[6] Heringstad B., Klemetsdal G., Ruane J. (2000). Selection for mastitis resistance in dairy cattle: A review with focus on the situation in the Nordic countries. Livest. Prod. Sci..

[7] Kibebew K. (2017). Bovine mastitis: A review of causes and epidemiological point of view. J. Biol. Agric. Healthc..

[8] Martin P., Barkema H., Brito L., Narayana S., Miglior F. (2018). Symposium review: Novel strategies to genetically improve mastitis resistance in dairy cattle. J. Dairy Sci..

[9] Hogan J.S., Smith K.L. Environmental streptococcal mastitis: Facts, fables, and fallacies. Proceedings of the Fifth International Dairy Housing Conference.

[10] Jain B., Tewari A., Bhandari B.B., Jhala M.K. (2012). Antibiotic resistance and virulence genes in Streptococcus agalactiae isolated from cases of bovine subclinical mastitis. Vet. Arhiv.

[11] Benić M., Maćešić N., Cvetnić L., Habrun B., Cvetnić Ž., Turk R., Đuričić D., Lojkić M., Dobranić V., Valpotić H. (2018). Bovine mastitis: A persistent and evolving problem requiring novel approaches for its control-a review. Vet. Arhiv.

[12] Botrel M.-A., Haenni M., Morignat E., Sulpice P., Madec J.-Y., Calavas D. (2009). Distribution and Antimicrobial Resistance of Clinical and Subclinical Mastitis Pathogens in Dairy Cows in Rhône-Alpes, France. Foodborne Pathog. Dis..

[13] Gomes F., Henriques M. (2016). Control of Bovine Mastitis: Old and Recent Therapeutic Approaches. Curr. Microbiol..

[14] Kaczorek E., Małaczewska J., Wójcik R., Rękawek W., Siwicki A.K. (2017). Phenotypic and genotypic antimicrobial susceptibility pattern of Streptococcus spp. isolated from cases of clinical mastitis in dairy cattle in Poland. J. Dairy Sci..

[15] Oliver S.P., Pighetti G.M. (2002). Mastitis Pathogens|Environmental Pathogens. Encycl. Dairy Sci..

[16] Wente N., Krömker V. (2020). Streptococcus dysgalactiae—Contagious or Environmental?. Animals.

[17] Raabe V.N., Shane A.L. (2019). Group B streptococcus (Streptococcus agalactiae). Gram Posit. Pathog..

[18] Evans J.J., Bohnsack J.F., Klesius P.H., Whiting A.A., Garcia J.C., Shoemaker C.A., Takahashi S. (2008). Phylogenetic relationships among Streptococcus agalactiae isolated from piscine, dolphin, bovine and human sources: A dolphin and piscine lineage associated with a fish epidemic in Kuwait is also associated with human neonatal infections in Japan. J. Med. Microbiol..

[19] Lyhs U., Kulkas L., Katholm J., Waller K.P., Saha K., Tomusk R.J., Zadoks R.N. (2016). Streptococcus agalactiae serotype IV in humans and cattle, northern Europe. Emerg. Infect. Dis..

[20] Pereira U., Mian G., Oliveira I., Benchetrit L., Costa G., Figueiredo H. (2010). Genotyping of Streptococcus agalactiae strains isolated from fish, human and cattle and their virulence potential in Nile tilapia. Vet. Microbiol..

[21] Phuektes P., Mansell P.D., Dyson R.S., Hooper N.D., Dick J.S., Browning G.F. (2001). Molecular epidemiology of Streptococcus uberis isolates from dairy cows with mastitis. J. Clin. Microbiol..

[22] Cvetnić L., Samardžija M., Habrun B., Kompes G., Benić M. (2016). Microbiological monitoring of mastitis pathogens in the control of udder health in dairy cows. Slov. Vet. Res..

[23] Hillerton J.E., Berry E.A. (2003). The management and treatment of environmental streptococcal mastitis. Vet. Clin. Food Anim. Pract..

[24] Krömker V., Reinecke F., Paduch J.-H., Grabowski N. (2014). Bovine Streptococcus uberis intramammary infections and mastitis. Clin. Microbiol. Open Access.

[25] Pitkälä A., Koort J., Björkroth J. (2008). Identification and antimicrobial resistance of Streptococcus uberis and Streptococcus parauberis isolated from bovine milk samples. J. Dairy Sci..

[26] Calvinho L.F., Almeida R.A., Oliver S.P. (1998). Potential virulence factors of Streptococcus dysgalactiae associated with bovine mastitis. Vet. Microbiol..

[27] Alnakip M.E.A., Rhouma N.R., Abd-Elfatah E.N., Quintela-Baluja M., Böhme K., Fernández-No I., Bayoumi M.A., Abdelhafez M.M., Taboada-Rodríguez A., Calo-Mata P. (2020). Discrimination of major and minor streptococci incriminated in bovine mastitis by MALDI-TOF MS fingerprinting and 16S rRNA gene sequencing. Res. Vet. Sci..

[28] Chaffer M., Friedman S., Saran A., Younis A. (2005). An outbreak of Streptococcus canis mastitis in a dairy herd in Israel. N. Z. Vet. J..

[29] Chen P., Qiu Y., Liu G., Li X., Cheng J., Liu K., Qu W., Zhu C., Kastelic J.P., Han B. (2021). Characterization of Streptococcus lutetiensis isolated from clinical mastitis of dairy cows. J. Dairy Sci..

[30] Hassan A.A., Akineden O., Usleber E. (2005). Identification of Streptococcus canis isolated from milk of dairy cows with subclinical mastitis. J. Clin. Microbiol..

[31] Król J., Twardoń J., Mrowiec J., Podkowik M., Dejneka G., Dębski B., Nowicki T., Zalewski W. (2015). Streptococcus canis is able to establish a persistent udder infection in a dairy herd. J. Dairy Sci..

[32] Tikofsky L., Zadoks R. (2005). Cross-infection between cats and cows: Origin and control of Streptococcus canis mastitis in a dairy herd. J. Dairy Sci..

[33] Erskine R.J., Wagner S., DeGraves F.J. (2003). Mastitis therapy and pharmacology. Vet. Clin. N. Am. Food Anim. Pract..

[34] Kalińska A., Gołębiewski M., Wójcik A. (2017). Mastitis pathogens in dairy cattle—A review. World Sci. News.

[35] Amin B., Deneke Y., Abdela N. (2017). Bovine Mastitis: Prevalence, Risk Factors and Isolation of Streptoccocus Species from Small Holders Dairy Farms in and Around Haramaya Town, Eastern Ethiopia. J. Med. Res..

[36] Dego O.K., Aral F. (2020). Current status of antimicrobial resistance and prospect for new vaccines against major bacterial bovine mastitis pathogens. Animal Reproduction in Veterinary Medicine.

[37] Oikonomou G., Machado V.S., Santisteban C., Schukken Y.H., Bicalho R.C. (2012). Microbial diversity of bovine mastitic milk as described by pyrosequencing of metagenomic 16s rDNA. PLoS ONE.

[38] Derakhshani H., Fehr K.B., Sepehri S., Francoz D., De Buck J., Barkema H.W., Plaizier J.C., Khafipour E. (2018). Invited review: Microbiota of the bovine udder: Contributing factors and potential implications for udder health and mastitis susceptibility. J. Dairy Sci..

[39] Falentin H., Rault L., Nicolas A., Bouchard D.S., Lassalas J., Lamberton P., Aubry J.-M., Marnet P.-G., Le Loir Y., Even S. (2016). Bovine Teat Microbiome Analysis Revealed Reduced Alpha Diversity and Significant Changes in Taxonomic Profiles in Quarters with a History of Mastitis. Front. Microbiol..

[40] Cole J.N., Pence M.A., Köckritz-Blickwede M.V., Hollands A., Gallo R.L., Walker M.J., Nizet V., Norrby-Teglund A., Low D.E. (2010). M Protein and Hyaluronic Acid Capsule Are Essential for In Vivo Selection of *covRS* Mutations Characteristic of Invasive Serotype M1T1 Group A *Streptococcus*. Mbio.

[41] Rajagopal L. (2009). Understanding the regulation of Group B Streptococcal virulence factors. Future Microbiol..

[42] Kell D.B., Heyden E.L., Pretorius E. (2020). The biology of lactoferrin, an iron-binding protein that can help defend against viruses and bacteria. Front. Immunol..

[43] O’Halloran F., Beecher C., Chaurin V., Sweeney T., Giblin L. (2016). Lactoferrin affects the adherence and invasion of Streptococcus dysgalactiae ssp. dysgalactiae in mammary epithelial cells. J. Dairy Sci..

[44] Almeida R.A., Dego O.K., Headrick S.I., Lewis M.J., Oliver S.P. (2015). Role of Streptococcus uberis adhesion molecule in the pathogenesis of Streptococcus uberis mastitis. Vet. Microbiol..

[45] Egan S.A., Ward P.N., Watson M., Field T.R., Leigh J.A. (2012). Vru (Sub0144) controls expression of proven and putative virulence determinants and alters the ability of Streptococcus uberis to cause disease in dairy cattle. Microbiology.

[46] Patel D., Almeida R.A., Dunlap J.R., Oliver S.P. (2009). Bovine lactoferrin serves as a molecular bridge for internalization of Streptococcus uberis into bovine mammary epithelial cells. Vet. Microbiol..

[47] Park H.-M., Almeida R., Luther D., Oliver S. (2002). Binding of bovine lactoferrin to Streptococcus dysgalactiae subsp. dysgalactiae isolated from cows with mastitis. FEMS Microbiol. Lett..

[48] Tong J., Sun M., Zhang H., Yang D., Zhang Y., Xiong B., Jiang L. (2020). Proteomic analysis of bovine mammary epithelial cells after in vitro incubation with S. agalactiae: Potential biomarkers. Vet. Res..

[49] Mamo W., Fröman G., Sundås A., Wadström T. (1987). Binding of fibronectin, fibrinogen and type II collagen to streptococci isolated from bovine mastitis. Microb. Pathog..

[50] Filippsen L.F. (1999). Bovine S protein (vitronectin) increases phagocytosis of Streptococcus dysgalactiae. Rev. Microbiol..

[51] Varhimo E., Varmanen P., Fallarero A., Skogman M., Pyörälä S., Iivanainen A., Sukura A., Vuorela P., Savijoki K. (2011). Alpha-and β-casein components of host milk induce biofilm formation in the mastitis bacterium Streptococcus uberis. Vet. Microbiol..

[52] Reinoso E.B. (2017). Bovine mastitis caused by streptococcus uberis. Virulence factors and biofilm. J. Microb. Biochem. Technol..

[53] Shukla R., Pandey V., Vadnere G.P., Lodhi S. (2019). Role of flavonoids in management of inflammatory disorders. Bioactive Food as Dietary Interventions for Arthritis and Related Inflammatory Disease.

[54] Alves-Barroco C., Roma-Rodrigues C., Balasubramanian N., Guimarães M.A., Ferreira-Carvalho B.T., Muthukumaran J., Nunes D., Fortunato E., Martins R., Santos-Silva T. (2019). Biofilm development and computational screening for new putative inhibitors of a homolog of the regulatory protein BrpA in Streptococcus dysgalactiae subsp. dysgalactiae. Int. J. Med. Microbiol..

[55] Emaneini M., Khoramian B., Jabalameli F., Abani S., Dabiri H., Beigverdi R. (2016). Comparison of virulence factors and capsular types of Streptococcus agalactiae isolated from human and bovine infections. Microb. Pathog..

[56] Mudzana R., Mavenyengwa R.T., Gudza-Mugabe M. (2021). Analysis of virulence factors and antibiotic resistance genes in group B streptococcus from clinical samples. BMC Infect. Dis..

[57] Loures R.A., de Pádua Pereira U., de Carvalho-Castro G.d.A., Mian G.F., da Costa Custódio D.A., da Silva J.R., da Costa G.M. (2017). Genetic diversity and virulence genes in Streptococcus uberis strains isolated from bovine mastitis. Semin. Ciências Agrárias.

[58] Lämmler C., Frede C. (1989). Binding of immunoglobulin G and albumin to Streptococcus dysgalactiae. Zent. Bakteriol..

[59] Jonsson H., Frykberg L., Rantamäki L., Guss B. (1994). MAG, a novel plasma protein receptor from Streptococcus dysgalactiae. Gene.

[60] Valentin-Weigand P., Traore M.Y., Blobel H., Chhatwal G.S. (1990). Role of α2-macroglobulin in phagocytosis of group A and C streptococci. FEMS Microbiol. Lett..

[61] Bobadilla F.J., Novosak M.G., Cortese I.J., Delgado O.D., Laczeski M.E. (2021). Prevalence, serotypes and virulence genes of Streptococcus agalactiae isolated from pregnant women with 35–37 weeks of gestation. BMC Infect. Dis..

[62] Pang M., Sun L., He T., Bao H., Zhang L., Zhou Y., Zhang H., Wei R., Liu Y., Wang R. (2017). Molecular and virulence characterization of highly prevalent Streptococcus agalactiae circulated in bovine dairy herds. Vet. Res..

[63] International Dairy Federation (2013). Guidelines for the Use and Interpretation of Bovine Milk Somatic Cell Count.

[64] Blagitz M.G., Souza F.N., Batista C.F., Azevedo L.F., Benites N.R., Melville P.A., Diniz S.A., Silva M.X., Haddad J.P., Heinnemann M.B. (2015). The neutrophil function and lymphocyte profile of milk from bovine mammary glands infected with Streptococcus dysgalactiae. J. Dairy Res..

[65] Beecher C., Daly M., Ross R.P., Flynn J., McCarthy T.V., Giblin L. (2012). Characterization of the bovine innate immune response in milk somatic cells following intramammary infection with Streptococcus dysgalactiae subspecies dysgalactiae. J. Dairy Sci..

[66] Florindo C., Barroco C.A., Silvestre I., Damião V., Gomes J.P., Spellerberg B., Santos-Sanches I., Borrego M.J. (2018). Capsular Type, Sequence Type and Microbial Resistance Factors Impact on DNase Activity of Streptococcus agalactiae Strains from Human and Bovine Origin. Eur. J. Microbiol. Immunol..

[67] Smeesters P.R., McMillan D.J., Sriprakash K.S. (2010). The streptococcal M protein: A highly versatile molecule. Trends Microbiol..

[68] Vasi J., Frykberg L., Carlsson L.E., Lindberg M., Guss B. (2000). M-like proteins of Streptococcus dysgalactiae. Infect. Immun..

[69] Duarte R.S., Bellei B.C., Miranda O.P., Brito M.A.V.P., Teixeira L.M. (2005). Distribution of antimicrobial resistance and virulence-related genes among Brazilian group B streptococci recovered from bovine and human sources. Antimicrob. Agents Chemother..

[70] Ashraf A., Imran M. (2018). Diagnosis of bovine mastitis: From laboratory to farm. Trop. Anim. Health Prod..

[71] Duarte C.M., Freitas P.P., Bexiga R. (2015). Technological advances in bovine mastitis diagnosis:an overview. J. Vet. Diagn. Investig..

[72] Keefe G. (2012). Update on control of Staphylococcus aureus and Streptococcus agalactiae for management of mastitis. Vet. Clin. Food Anim. Pract..

[73] Damm M., Holm C., Blaabjerg M., Bro M.N., Schwarz D. (2017). Differential somatic cell count—A novel method for routine mastitis screening in the frame of Dairy Herd Improvement testing programs. J. Dairy Sci..

[74] Wethal K., Svendsen M., Heringstad B. (2020). A genetic study of new udder health indicator traits with data from automatic milking systems. J. Dairy Sci..

[75] Viguier C., Arora S., Gilmartin N., Welbeck K., O’Kennedy R. (2009). Mastitis detection: Current trends and future perspectives. Trends Biotechnol..

[76] Mansor R., Mullen W., Albalat A., Zerefos P., Mischak H., Barrett D.C., Biggs A., Eckersall P.D. (2013). A peptidomic approach to biomarker discovery for bovine mastitis. J. Proteom..

[77] Tenhagen B.-A., Köster G., Wallmann J., Heuwieser W. (2006). Prevalence of mastitis pathogens and their resistance against antimicrobial agents in dairy cows in Brandenburg, Germany. J. Dairy Sci..

[78] Bonsaglia E.C.R., Gomes M.S., Canisso I.F., Zhou Z., Lima S.F., Rall V.L.M., Oikonomou G., Bicalho R.C., Lima F.S. (2017). Milk microbiome and bacterial load following dry cow therapy without antibiotics in dairy cows with healthy mammary gland. Sci. Rep..

[79] Gill J.J., Pacan J.C., Carson M.E., Leslie K.E., Griffiths M.W., Sabour P.M. (2006). Efficacy and pharmacokinetics of bacteriophage therapy in treatment of subclinical Staphylococcus aureus mastitis in lactating dairy cattle. Antimicrob. Agents Chemother..

[80] O’flaherty S., Coffey A., Meaney W., Fitzgerald G., Ross R. (2005). Inhibition of bacteriophage K proliferation on Staphylococcus aureus in raw bovine milk. Lett. Appl. Microbiol..

[81] Radzikowski D., Kalińska A., Ostaszewska U., Gołębiewski M. (2020). Alternative solutions to antibiotics in mastitis treatment for dairy cows-a review. Anim. Sci. Pap. Rep..

[82] Yang X., Ouyang W., Sun J., Li X. (2009). Post-antibiotic effect of Amoxicillin nanoparticles against main pathogenic bacteria of Bovine mastitis in vitro. J. Northwest A F Univ. Nat. Sci. Ed..

[83] Serna-Cock L., Enríquez-Valencia C.E., Jiménez-Obando E.M., Campos-Gaona R. (2012). Effects of fermentation substrates and conservation methods on the viability and antimicrobial activity of Weissella confusa and its metabolites. Electron. J. Biotechnol..

[84] Wu J., Hu S., Cao L. (2007). Therapeutic effect of nisin Z on subclinical mastitis in lactating cows. Antimicrob. Agents Chemother..

[85] Abd El Hafez S.M., Ismael A.B., Mahmoud M.B., Elaraby A.K.A. (2013). Development of new strategy for non-antibiotic therapy: Bovine lactoferrin has a potent antimicrobial and immunomodulatory effects. Adv. Infect. Dis..

[86] Kutila T., Pyörälä S., Kaartinen L., Isomäki R., Vahtola K., Myllykoski L., Saloniemi H. (2003). Lactoferrin and Citrate Concentrations at Drying-off and During Early Mammary Involution of Dairy Cows. J. Vet. Med. Ser. A.

[87] Malinowski E., Niewitecki W., Nadolny M., Lassa H., Smulski S. (2006). Effect of lysozyme dimer injections on results of intramammary treatment of acute mastitis in cows. Med. Weter..

[88] Ananda Baskaran S., Kazmer G.W., Hinckley L., Andrew S.M., Venkitanarayanan K. (2009). Antibacterial effect of plant-derived antimicrobials on major bacterial mastitis pathogens in vitro. J. Dairy Sci..

[89] De Barros M., Perciano P.G., Dos Santos M.H., De Oliveira L.L., Costa É.D.M., Moreira M.A.S. (2017). Antibacterial Activity of 7-Epiclusianone and Its Novel Copper Metal Complex on Streptococcus spp. Isolated from Bovine Mastitis and Their Cytotoxicity in MAC-T Cells. Molecules.

[90] Fonseca A.P., Estrela F.T., Moraes T.S., Carneiro L.J., Bastos J.K., Santos R.A.d., Ambrósio S.R., Martins C.H.G., Veneziani R.C.S. (2013). In Vitro Antimicrobial Activity of Plant-Derived Diterpenes against Bovine Mastitis Bacteria. Molecules.

[91] Gopinath S.M., Suneetha T.B., Mruganka V.D., Ananda S. (2011). Evaluation of antibacterial activity of Tabernaemontana divaricata (L.) leaves against the causative organisms of bovine mastitis. Int. J. Res. Phytochem. Pharmacol..

[92] Laloučková K., Malá L., Slaničková P., Skřivanová E. (2019). In vitro antimicrobial effect of palm oils rich in medium-chain fatty acids against mastitis-causing Gram-positive bacteria. Czech J. Anim. Sci..

[93] Montironi I.D., Cariddi L.N., Reinoso E.B. (2016). Evaluation of the antimicrobial efficacy of Minthostachys verticillata essential oil and limonene against Streptococcus uberis strains isolated from bovine mastitis. Rev. Argent. Microbiol..

[94] Krömker V., Leimbach S. (2017). Mastitis treatment—Reduction in antibiotic usage in dairy cows. Reprod. Domest. Animals.

[95] Barlow J. (2011). Mastitis therapy and antimicrobial susceptibility: A multispecies review with a focus on antibiotic treatment of mastitis in dairy cattle. J. Mammary Gland Biol. Neoplasia.

[96] Kateete D.P., Kabugo U., Baluku H., Nyakarahuka L., Kyobe S., Okee M., Najjuka C.F., Joloba M.L. (2013). Prevalence and antimicrobial susceptibility patterns of bacteria from milkmen and cows with clinical mastitis in and around Kampala, Uganda. PLoS ONE.

[97] Makovec J.A., Ruegg D.P.L. (2003). Antimicrobial resistance of bacteria isolated from dairy cow milk samples submitted for bacterial culture: 8905 samples (1994–2001). J. Am. Vet. Med. Assoc..

[98] Persson Y., Nyman A.-K.J., Grönlund-Andersson U. (2011). Etiology and antimicrobial susceptibility of udder pathogens from cases of subclinical mastitis in dairy cows in Sweden. Acta Vet. Scand..

[99] Rato M.G., Bexiga R., Florindo C., Cavaco L.M., Vilela C.L., Santos-Sanches I. (2013). Antimicrobial resistance and molecular epidemiology of streptococci from bovine mastitis. Vet. Microbiol..

[100] Rossitto P., Ruiz L., Kikuchi Y., Glenn K., Luiz K., Watts J., Cullor J.S. (2002). Antibiotic susceptibility patterns for environmental streptococci isolated from bovine mastitis in central California dairies. J. Dairy Sci..

[101] Thomas V., de Jong A., Moyaert H., Simjee S., El Garch F., Morrissey I., Marion H., Vallé M. (2015). Antimicrobial susceptibility monitoring of mastitis pathogens isolated from acute cases of clinical mastitis in dairy cows across Europe: VetPath results. Int. J. Antimicrob. Agents.

[102] Boireau C., Cazeau G., Jarrige N., Calavas D., Madec J.-Y., Leblond A., Haenni M., Gay É. (2018). Antimicrobial resistance in bacteria isolated from mastitis in dairy cattle in France, 2006–2016. J. Dairy Sci..

[103] Minst K., Märtlbauer E., Miller T., Meyer C. (2012). Streptococcus species isolated from mastitis milk samples in Germany and their resistance to antimicrobial agents. J. Dairy Sci..

[104] Burović J. (2020). Isolation of bovine clinical mastitis bacterial pathogens and their antimicrobial susceptibility in the Zenica region in 2017. Vet. Stanica.

[105] Haenni M., Lupo A., Madec J.-Y. (2018). Antimicrobial Resistance in *Streptococcus* spp. ASM J..

[106] Pitkälä A., Haveri M., Pyörälä S., Myllys V., Honkanen-Buzalski T. (2004). Bovine mastitis in Finland 2001—prevalence, distribution of bacteria, and antimicrobial resistance. J. Dairy Sci..

[107] Zadoks R., Allore H., Barkema H., Sampimon O., Wellenberg G., Gröhn Y., Schukken Y. (2001). Cow-and quarter-level risk factors for Streptococcus uberis and Staphylococcus aureus mastitis. J. Dairy Sci..

[108] Leigh J., Finch J., Field T., Real N., Winter A., Walton A., Hodgkinson S. (1999). Vaccination with the plasminogen activator from Streptococcus uberis induces an inhibitory response and protects against experimental infection in the dairy cow. Vaccine.

[109] Finch J.M., Hill A.W., Field T.R., Leigh J.A. (1994). Local vaccination with killed Streptococcus uberis protects the bovine mammary gland against experimental intramammary challenge with the homologous strain. Infect. Immun..

[110] Collado R., Montbrau C., Sitjà M., Prenafeta A. (2018). Study of the efficacy of a Streptococcus uberis mastitis vaccine against an experimental intramammary infection with a heterologous strain in dairy cows. J. Dairy Sci..

[111] Fontaine M.C., Perez-Casal J., Song X.-M., Shelford J., Willson P.J., Potter A.A. (2002). Immunisation of dairy cattle with recombinant Streptococcus uberis GapC or a chimeric CAMP antigen confers protection against heterologous bacterial challenge. Vaccine.

